# Maize Cropping Systems Mapping Using RapidEye Observations in Agro-Ecological Landscapes in Kenya

**DOI:** 10.3390/s17112537

**Published:** 2017-11-03

**Authors:** Kyalo Richard, Elfatih M. Abdel-Rahman, Sevgan Subramanian, Johnson O. Nyasani, Michael Thiel, Hosein Jozani, Christian Borgemeister, Tobias Landmann

**Affiliations:** 1 International Center for Insect Physiology and Ecology (ICIPE), P.O. Box 30772, 00100 Nairobi, Kenya; rkyalo@icipe.org (K.R.); eabdel-rahman@icipe.org (E.M.A.-R.); ssubramania@icipe.org (S.S.); Johnson.Nyasani@kalro.org (J.O.N.); 2 Department of Agronomy, Faculty of Agriculture, University of Khartoum, Khartoum North 13314, Sudan; 3 Crop Health Unit, Kenya Agricultural and Livestock Research Organization, Embu Research Centre, P.O. Box 27, 60100 Embu, Kenya; 4 Department of Remote Sensing, University of Würzburg, Oswald-Külpe-Weg 86, 97074 Würzburg, Germany; michael.thiel@uni-wuerzburg.de (M.T.); hoseinjafary@aol.com (H.J.); 5 Center for Development Research (ZEF), Department of Ecology and Natural Resources Management, University of Bonn, Walter-Flex-Str. 3, 53113 Bonn, Germany; cb@uni-bonn.de

**Keywords:** RapidEye, bi-temporal, cropping systems, random forest, Kenya

## Abstract

Cropping systems information on explicit scales is an important but rarely available variable in many crops modeling routines and of utmost importance for understanding pests and disease propagation mechanisms in agro-ecological landscapes. In this study, high spatial and temporal resolution RapidEye bio-temporal data were utilized within a novel 2-step hierarchical random forest (RF) classification approach to map areas of mono- and mixed maize cropping systems. A small-scale maize farming site in Machakos County, Kenya was used as a study site. Within the study site, field data was collected during the satellite acquisition period on general land use/land cover (LULC) and the two cropping systems. Firstly, non-cropland areas were masked out from other land use/land cover using the LULC mapping result. Subsequently an optimized RF model was applied to the cropland layer to map the two cropping systems (2nd classification step). An overall accuracy of 93% was attained for the LULC classification, while the class accuracies (PA: producer’s accuracy and UA: user’s accuracy) for the two cropping systems were consistently above 85%. We concluded that explicit mapping of different cropping systems is feasible in complex and highly fragmented agro-ecological landscapes if high resolution and multi-temporal satellite data such as 5 m RapidEye data is employed. Further research is needed on the feasibility of using freely available 10–20 m Sentinel-2 data for wide-area assessment of cropping systems as an important variable in numerous crop productivity models.

## 1. Introduction

Agro-ecological systems in Africa are particularly vulnerable to climate variability and climate change due to their over dependence on rainfall [1]. The particular cropping system used by farmers is often a key determinant in climate-smart agriculture concepts, crop diversification and livelihoods strategies [2]. While information about cropland extents or crop acreages is increasingly available and being used in food supply projections [3], explicit information about the actual cropping systems is not largely utilized or available. This leads to significant uncertainties in crop production models and ultimately in food security projections for Africa [4].

The cropping system is defined as the planting sequence of crops applied to an agricultural area or field over a certain period. In agronomical terms, an agricultural field can be mono-cropped, inter-cropped, relay cropped, mixed-cropped or under crop rotation (i.e., planting different crops in sequential years) [5]. Mixed-cropping is a common practice on small-scale farms in developing countries like Kenya. In Kenya, maize (*Zea mays* L.) is the staple food, and it is common to find it mixed with bean (*Phaseolus vulgaris* L.) [6]. The degree of mixed cropping is often determined by the need for diversification against a backdrop of increased climate variability and the need to increase soil fertility and soil moisture regimes to sustain or increase crop productivity [7]. In this study, we define mixed cropping specifically as maize grown in a spatial arrangement with other leguminous crops on the same field within the same growing season and mono-cropping as maize grown as a single crop within the same time frame and field.

High and medium spatial resolution satellite data have been widely used for agricultural land use mapping in different agro-ecological zones in Africa and beyond [8,9]. However, many studies alluded to the challenges of accurately mapping crops and cropping systems in Africa on a landscape scale primarily due to the small scale and highly fragmented nature of cropping patterns as well as their intra- and inter-annual dynamics [10]. The temporal and spatial high variability of cropping systems is often a result of incoherent farmers decisions (the planting date often varies from one season or year to the next) and other localized and hard to quantify socio-economic factors [11]. Moreover, rain fed crops are largely indiscriminate from some natural vegetation communities such as grassland during the wet or growing season when both (the crops and some natural vegetation types) have the same phenological growing cycles [12]. Thus, landscape scale crop mapping mechanisms using medium resolution data such as 30 m Landsat, a frequently used type of dataset for crop mapping in Africa [13], has resulted in high spectral heterogeneity and poor mapping results [14]. Essentially, remotely sensed data can provide spatially coherent information only on crop acreage and crop vitality on landscape scales with an advantage over traditional conventional surveying methods that are often tedious and costly (ineffective), especially if crop assessments are performed over larger areas [15].

Relatively newly available 5 m RapidEye data are suitable for crop mapping in highly fragmented and dynamic landscapes because of the higher and enhanced geometrical resolution of the satellite system, particularly in small-scale farming systems in Africa where the size of field is relatively small (≤1.25 ha) [8,10,16]. The enhanced spectral resolution of RapidEye data in the red-edge waveband domain, for instance, allows for significantly enhanced land use classification and improved crop discrimination. This could be due to strong correlations between the vegetation spectral features at the red-edge band and chlorophyll content, and the sensitivity of the red-edge band to differences in leaf structure [17,18,19]. Combined with state-of-the-art and hierarchical classification approaches using robust machine learning classification algorithms and RapidEye data from different time steps, including their derived vegetation indices, explicit and permissible accurate crop type mapping results even in complex African landscapes can be generated [8,10]. Various types of non-parametric machine learning classification methods like random forest (RF) have been successfully applied to mapping crops in Africa [8,10].

Mulianga et al. [20] characterized cropping practices (crop type and harvest mode) of sugarcane-based cropping systems in Kenya using a resampled 15 m multi-temporal Landsat dataset and a maximum likelihood classifier. However, sugarcane is usually grown on large-scale commercial and homogeneous fields that can be easily discriminated [21]. To the best of our knowledge, no study has yet attempted to map maize-cropping systems in heterogeneous landscapes in Africa and, moreover, no study is known that utilized RapidEye time-series data using a machine learning classification approach in this regard. Accordingly, the main objective of the study was to examine the utility of random forest (RF) classifier and new-generation RapidEye imagery with enhanced waveband coverage in the red-edge spectral region for cropping systems mapping. Specifically, we aimed to develop a (semi-automated) processing scheme to find the optimal RF model parameters by analyzing the relative model contribution of the RapidEye spectral indices and individual waveband regions (bands) for crop systems mapping. Having information on the spatial distributions of maize systems would ultimately help to better understand factors that contribute to crop productivity and farm or field level yield variability [22].

## 2. Study Area

**Figure 1:**
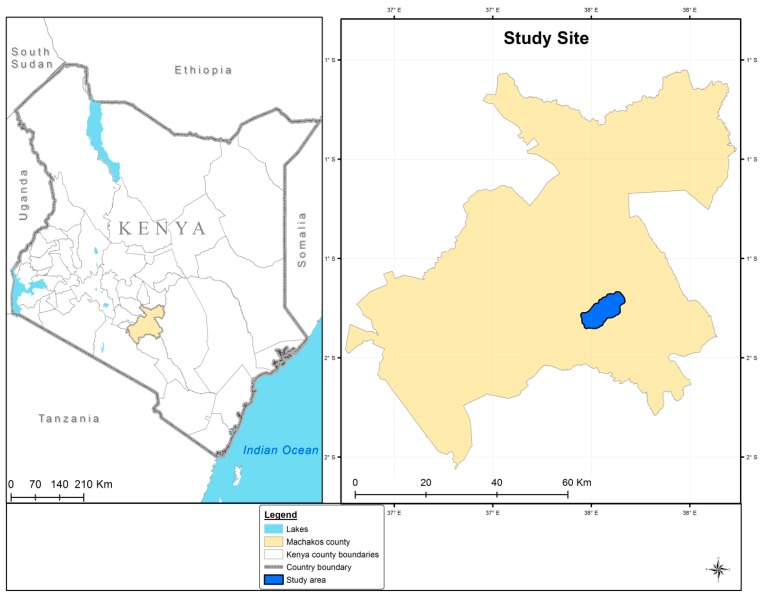
Locations of the study area in Machakos County, Kenya, with the dark blue polygon showing the study area. Grey lines illustrate sub-county boundaries.

The study area is in Machakos County, about 100 km south-east of Nairobi in Kenya (Figure 1). The study area lies between the latitudes 1°17′53.71″ S and 1°31′8.54″ S and between the longitudes of 37°28′15.79″ E and 37°40′33.43″ E. The total study area covers about 677 km^2^ with elevation ranging from 400 m to 2100 m above mean sea level (MAMSL). The climate is semi-arid with a highly variable rainfall regime distributed over two rainy seasons, hence two cropping seasons namely the short rain season and the long rain season. Short rains occur from November to January, and long rains from March to June with an average rainfall ranging from 500 to 2000 mm (mean annual precipitation) and a mean annual maximum temperature of 28 °C [23].

The most widespread vegetation type in Machakos is semi-arid deciduous thicket and bush land, dominated by *Acacia* spp. (Fabaceae) and *Commiphora* spp. (Burseraceae). In drier locations below the elevation of 900 m, thorn bush grades into semi-desert vegetation. Moreover, arable land covers about 64% of the total landmass of the study area [24], with mixed cropping regularly practiced in this region [11]. The most prevalent crops in the region are maize, bean, pigeon pea and cowpea. Maize and bean in most cases are mixed in the long rainy season, while cowpea is mainly mixed with maize and bean in the short rainy season. Recent uncertainties in rainfall patterns have encouraged mixed cropping with the majority of farmers mixing maize with bean [25]. In addition, irrigated farming is also practiced in locations neighboring the Athi River to facilitate small-scale cultivation of vegetables, tomatoes and chili peppers.

## 3. Methodology

**Figure 2:**
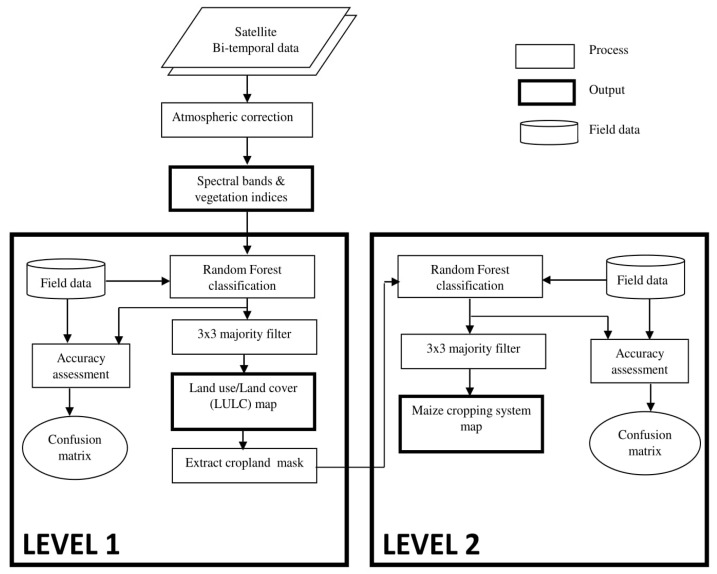
Flow chart of the hierarchical classification approach using random forest (RF) classifier and Bi-temporal RapidEye data.

Figure 2 summarizes our methodological approach for mapping the two cropping systems (mono- and mixed cropping). Basically, we employed a 2-step hierarchical classification approach to map the maize cropping systems using bi-temporal RapidEye data and the RF classification algorithm. In the first step, we produced a general land use/land cover (LULC) classification map to separate cropland from non-cropland in order to reduce the data complexity for subsequent classification [10]. In the second step, we classified the extracted crop mask into two cropping systems (*viz*. mono- and mixed cropping).

### 3.1. Image Acquisition and Preprocessing

Two RapidEye images were acquired for the study area on the 3 January 2015 and the 27 January 2015, during the maize stem elongation (RE1) and flowering (RE2) crop phenological development stages, respectively. These two maize phenological development stages are characterized using the BBCH (Biologische Bundesanstalt, Bundessortenamt und Chemische Industrie) scale [26]. The images were acquired at two different acquisition windows to assess the effect of crop phenology (the crop life cycle) on mapping cropping systems with an assumption that the spectral features of the crops (mainly bean and cowpea) that are mainly planted with maize are distinguishable during the maize flowering stage.

RapidEye provides images with spatial resolution of 5 m and five spectral bands (wavelength regions) which are located at blue (440–550 nm), green (520–590 nm), red (630–685 nm), red-edge (690–730 nm) and near infrared (760–850 nm) regions of the electromagnetic spectrum. The RapidEye Ortho product (Level 3A) was utilized. To retrieve surface reflectance, atmospheric correction was applied using the atmospheric-topographic correction (ATCOR3) software to remove haze and other atmospheric interferences. ATCOR3 is an extension of model ATCOR2 that permits extended three-dimensional topographic corrections by inclusion of digital elevation model (DEM) data to remove illumination difference due to topography effects [27]. Parameters, such as satellite azimuth, illumination elevation, azimuth and incidence angle, which are used for atmospheric corrections, were obtained from respective metadata files for each image. To reduce illumination effects caused by the terrain on RapidEye imagery, topographic corrections were performed using Shuttle Radar Topographic Mission (SRTM) digital elevation (DEM) data. The 30 m SRTM DEM data, used in topographic correction processes, were re-sampled to 5 m pixel resolution using a bilinear interpolation technique. Due to different date and time acquisitions of each tile in the RapidEye mosaic, a tile specific stepwise normalization technique using multivariate alteration detection (IMAD) was used to normalize the tiles via a central normalization reference tile. The image data sets were geo-referenced to Universal Transverse Mercator (UTM, zone 36 south).

Subsequently, mosaicking was applied on all the co-registered normalized tiles and two mosaics having a size of ~61 by 61 km each were produced initially. To align all the corresponding pixels, the two mosaicked images were co-registered to each other using image-to-image co-registration to ascertain the alignment of corresponding pixels. Finally, regions that were covered by clouds have been masked out. We utilized 30 vegetation indices calculated from each RapidEye data set (Table 1) together with the respective five RapidEye bands as input into the RF classification algorithm. The inclusion of vegetation indices that are related to vegetation biochemical and biophysical traits like chlorophyll activity and leaf area index, together with the individual spectral bands, has proved to significantly improve crop classification accuracies in heterogeneous landscapes [10].

### 3.2. Field Data Collection

A field campaign was conducted within three days from the first image acquisition date (3 January 2015) to collect reference data on croplands which in our study area are solely mono- and mixed maize cropping systems. Furthermore, field data were collected on non-cropland, composed of water bodies, artificial surfaces and natural vegetation. A stratified random sampling was followed to collect the reference data. A handheld Global Positioning System (GPS) device with an error of ±3 m was used to locate the reference control points. Once a field was identified, we delineated the field boundaries (polygon) within a minimum area of 30 by 30 m. To avoid the edge effect, we collected the polygon data five meters away from the edge of each field. Geo tagged photographs of each cropping system in the sample fields were taken from the main four cardinal directions and from the center of the fields for further inspections of the cropping systems and crop age. To mitigate the effect of soil background on the crops’ spectral features, we only sampled maize fields (mono- and mixed cropping systems) that were about three weeks old at the first image acquisition date. The reference data were randomly divided into 70% training and 30% validation set. The training set was used to train the RF classifier while the validation dataset was used to evaluate the accuracy.

### 3.3. Variable Importance Measure and Classification

A supervised machine learning RF classification algorithm [44] was used to classify the bi-temporal RapidEye image data. RF is considered a robust and efficient classification approach for crop mapping using high spatial resolution satellite data like RapidEye, especially within heterogeneous landscape [10]. It has the potential to handle noisy and highly correlated predictor variables, which commonly occur in remotely sensed data [45]. In particular, RF is an ensemble modeling technique, developed by Liaw and Wiener [46], to improve the classification and regression trees (CART) by combining a large set of decision trees. Each tree in the RF ensemble is built from a bootstrapped random sample containing approximately two-thirds of the training data drawn at random with replacement. The remaining one-third of the data that is not included in the bootstrapped training sample, i.e., the out-of-bag (OOB) samples, is used to internally evaluate the classification performance. The RF classifier uses two user-defined parameters (*ntree* and *mtry*). To improve the classification accuracy, the number of trees (*ntree*) grown and variables used at each tree split (*mtry*) were optimized based on the OOB error rate with a grid search and a tenfold cross validation method [47]. The number of optimal trees (*ntree*) was searched between 500 to 2500 using a 500 interval, while the optimal *mtry* was searched on the *mtry* vector of a multiplicative factor with the default *mtry* being the square root of the total number of spectral variables (indices and/or bands) [48]. The ensemble measures the importance of each spectral variable used in the classification by utilizing the permutation of variables which calculates variable importance as the mean decrease in classification accuracy using the OOB samples.

To select the optimal combination of spectral variables that achieved significant accuracies from the important variables returned by the RF classification model using the OOB error rate, we used the RF backward feature elimination method using the “varSelRF” package [49] in the R statistical software [50] for level 2 of distinguishing the two maize cropping systems. To select the most relevant spectral variables without any over-fitting, a .632+ bootstrap method with a leave-one-out cross-validation procedure and replacement from samples that are not part of the RF classification was applied [51]. The optimum numbers of spectral variables selected were employed to produce the final cropping systems map. Finally, a 3 × 3 post-classification majority filter was applied to spatially smooth the classified images’ dominant classes so as to reduce salt-and-pepper effects in the classification output map.

### 3.4. Accuracy Assessment

A confusion matrix was constructed to assess the accuracy of the classified maps using the overall accuracy (OA), producers’ accuracy (PA) and users’ accuracy (UA). The most recently proposed allocation quantity (QD) and allocation (AD) disagreements [52] were also calculated from the classification confusion matrix to evaluate the reliability of the classification map and to measure the agreement between the predicted classification features and the reference field data (OOB samples). Class-wise accuracy assessment was performed for each class using F1-score [18]. This measure represents the harmonic mean between PA and UA for each class i as follows:

(1) $${\left(F1\right)}_{i}=\frac{2\times {\mathrm{PA}}_{i}\times {\mathrm{UA}}_{i}}{{\mathrm{PA}}_{i}+{\mathrm{UA}}_{i}}$$

The advantage of using the F1-score for class accuracy evaluation is to give equal importance to both precision and recall, by combining PA and UA into a fused measure.

## 4. Results

### 4.1. Parameterization of the Random Forest Classifiers

**Figure 3:**
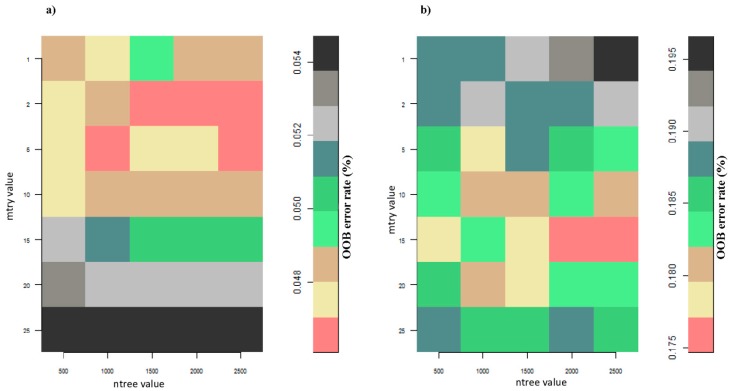
Random forest (RF) *mtry* and *ntree* optimization grid for the land use land cover (LULC) classification result (**a**) and for the mono- and mixed maize cropping systems mapping result (**b**) using the internal out-of-bag (OOB) error rate of RF resulting from a grid search with a tenfold cross validation setting.

The RF grid search with the tenfold cross validation method indicated that *ntree* value of 1,000 combined with *mtry* value of 5 was optimal for classifying general land use and land cover (LULC) classes (Figure 3a) to separate cropland from non-cropland. On the other hand, the *mtry* value of 15 combined with *ntree* value of 2000 resulted in the lowest OOB error rate of 0.18% for classifying the mono- and mixed maize cropping systems as shown (Figure 3b).

### 4.2. Spectral Variable Importance for Crop Systems Mapping

**Figure 4:**
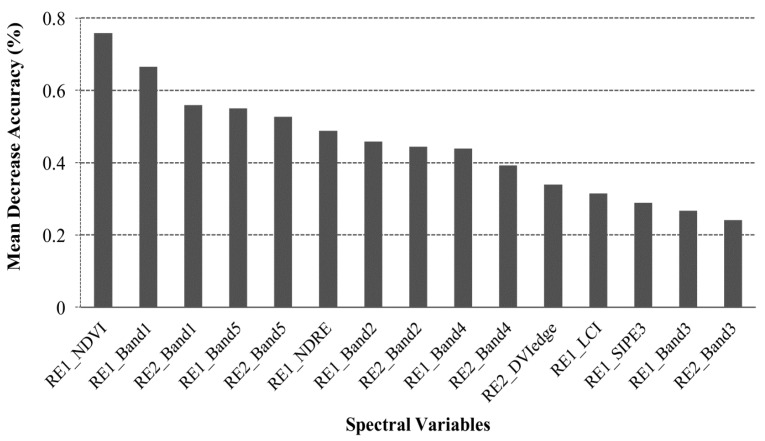
Mean decrease in accuracy of the 15 most important input variables that were selected using the random forest backward feature elimination function and the .632+ bootstrapping function on importance ranking.

The backward variable selection method, applied on the RF variable importance ranking, resulted in selecting 15 RapidEye spectral variables (Figure 4) that were found to be the most relevant for mapping mono- and mixed maize cropping systems after crop masking. Using the LULC map result (Figure 2), nine and six spectral variables, respectively, were selected as important variables from the two RapidEye images, captured during the stem elongation and the flowering development stages, respectively (Figure 4). Moreover, most of the selected variables from both observation periods were the RapidEye spectral wavebands themselves. In general, all five RapidEye bands (blue, green, red, red-edge and near infrared) were selected as useful spectral features for classifying the two maize cropping systems, while only five indices (RE1_NDVI, RE1_NDRE, RE2_DVIedge, RE1_LCI and RE1_SIPE3) were useful for separating different maize cropping systems (Figure 4).

### 4.3. Maize Cropping Systems Mapping

**Figure 5:**
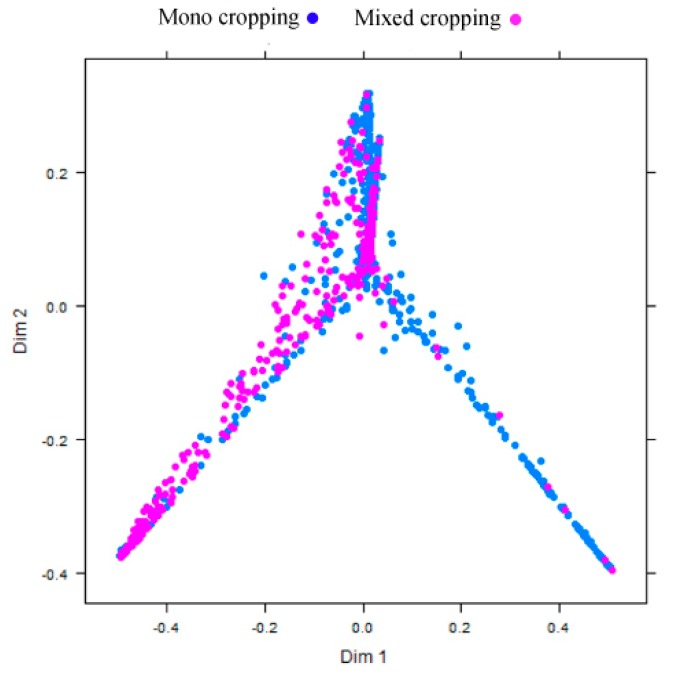
Random forest class separability proximity matrix using multidimensional scaling (MDS); Dim 1 refers to dimension 1 and Dim 2 to dimension 2.

Visual interpretation of the RF capability to separate the two mapped classes (mono cropping and mixed cropping) using a multidimensional class separability proximity matrix indicate that the majority of the pixel are generally well separable as shown in Figure 5.

**Figure 6:**
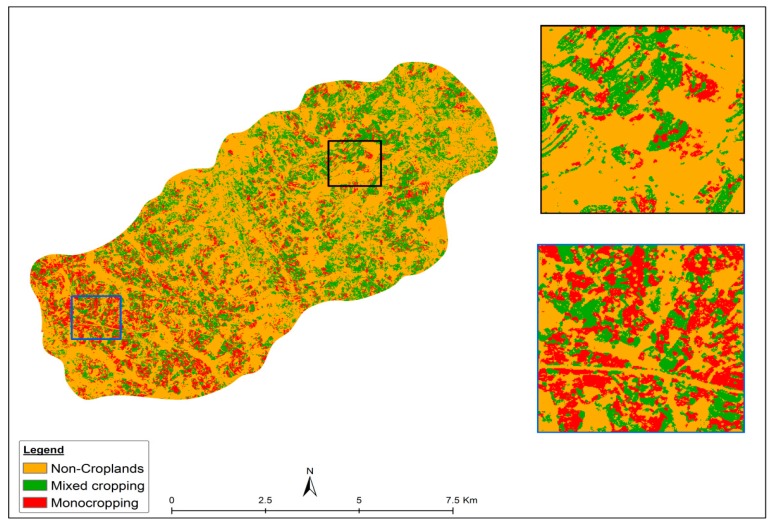
Maize cropping systems classification map obtained using the proposed classification scheme (Figure 2). The two inserts, as black and blue squares, illustrate two contrasting areas in terms of cropping systems.

The final thematic cropping systems map produced via the RF algorithm is shown in Figure 6. It shows that mixed-cropped fields are mostly present in the middle and towards the north-eastern part of the study area which is characterized by a lower altitude (around 1100 MAMSL), while most of the mono-cropped fields are found in the south-western part of the study area at a mean altitude of 2000 MAMSL Mono cropped fields in the higher lying areas in the range of 1400–2000 m above sea level appeared larger and less scattered (fragmented) than the mixed cropped fields in the lower areas with elevation below 1400 m (Figure 6).

### 4.4. Classification Accuracies

To understand the accuracies of the cropping systems map (level 2 classification) that uses the cropland mask extracted from LULC result (level 1 classification), the accuracies of LULC map are herein reported. The main results of the accuracy assessment for the hierarchical level 1 LULC classification are summarized in Table 2 for the three different combinations which included the RE bands, the RE band with all vegetation indices and RF selected spectral variables (bands and vegetation indices). The most accurate LULC mapping result was obtained from the most important selected vegetation indices and spectral bands listed in Figure 4 using RF backward selection criteria with an overall accuracy of 93.2% and a kappa coefficient of 0.91. Table 3 presents the per-pixel evaluation confusion matrix for the LULC map. Individual accuracies (PA and UA) were consistently over 87% with the F1-score averagely above 0.88 for all classes (Table 3). This suggests a very good concealment of croplands from other LULC classes regardless of cropland being slightly confused with the natural vegetation class.

Table 4 summarizes classification accuracies for three classifications calibrated for mapping the two maize cropping systems (level 2 classification). The optimized RF cropping system mapping result using only the most relevant spectral variables selected by RF gave an overall accuracy of 85.7% (kappa coefficient of 0.84) whereas the non-optimized result, using all RapidEye bands and the vegetation indices, gave a lower overall accuracy of 73.4%. In addition, individual PA and UA for the optimized RF result (Table 5) were consistently above 84% for both classes with a low QD score of 1% and a relatively high AD score of 13% for both cropping systems, respectively.

## 5. Discussion

The classification results from this study demonstrated the usefulness of the bi-temporal RapidEye imagery and RF classification tool for mapping the two major maize cropping systems in heterogeneous agro-ecological landscapes. This demonstrates the capability of high spatial resolution data with better spectral coverage such as RapidEye to distinguish different cropping systems, given both the size and shape of fields in African agro-ecological systems. The two images could capture the spectral (phenological) profiles of the two cropping systems thus resulting in a better discriminatory power between the two cropping systems [53]. In addition, RF selected NDVI from the first RapidEye acquisition (RE1) among the most significant variable in separating the two cropping systems. This could be due to the fact that at the stem elongation phenological development stage, mono- and mixed cropping systems are easily distinguishable, while at the flowering phenological growth stage the two cropping systems seem to have similar morphological and spectral properties [54]. We observed that the most important spectral indices selected by RF from the two acquisitions are commonly related to plant biophysical properties such as Leaf Area Index (LAI) and net primary productivity [55]. It is expected that mono- and mixed maize cropping systems can differ considerably in these traits since mono-cropped maize is known to exhibit a lower LAI, especially during the flowering stage, than maize mixed with legumes (among other factors, largely due to the absence of bare soil) [56]. It is interesting to note that the RE2_NDVI was not amongst the selected most important variables. That could be due to fact that the second RE2 acquisition corresponded to the maize flowering stage in which the spectral contributions are not overly characterized by chlorophyll activities (NDVI) but more by spectral contributions from non-chlorophyll plant components, i.e., the cobs, wilting leaves and the maize flowers. Moreover, the RF backward variable selection process showed that the inclusion of the red-edge bands and spectral indices that use the red-edge bands (i.e., DVI-edge) were relevant for the maize cropping systems mapping result. Similarly, Schuster et al. [18] found that the red-edge bands improved land use classification by 2.7%.

The spatial cropping system differences we observed between the low and high altitude areas (Figure 6) could be due to more favorable climatic conditions for crop production within mountainous regions as single (mono) crop production is more feasible in upland areas that receive higher rainfall [57]. Farmers in drier areas often opt to combine maize with leguminous crops on the same field (mixed cropping) to improve soil fertility and soil moisture in order to attain permissible yields [58]. The crop systems patterns also showed considerable differences in field size between the lower and higher lying areas within the study area (approximately 0.8 ha in the low altitudinal areas versus an average field size of 0.2 ha in the higher lying areas). These field size differences could be confirmed from the field observations.

The crop system mapping results indicated comparatively high OA of 85.7% and individual class accuracies (PA and UA) > 84% [59]. The high accuracies could be due to, primarily, the optimal acquisition dates of the imagery, i.e., during the stem elongation and flowering crop development stages, respectively. These critical crop stages are known to produce better separation between agricultural fields and surrounding natural vegetation [60]. Specific confusions between the two cropping system classes could have numerous reasons such as heterogeneity of the landscape, variation in crop age (planting dates) and other agronomical practices (e.g., ploughing) [10,61]. Field heterogeneity is largely affected by within field spectral variations that are larger than inter-field spectral variations due to the crop morphological and physiological properties [62]. This confusion is exacerbated by the amount of weed infestation per cropped area where a weed-infested mono-cropped maize field could exhibit a similar spatial arrangement, and thus spectral response, to a maize field inter-cropped with a legume like cowpea or bean [63]. Some sample mono-cropped fields in our study area were badly managed and infested by weeds that could have caused the spectral confusion with inter-cropped fields as previously mentioned. Another reason for heterogeneity and spectral confusion could be the fact that some farmers maintain trees within their fields and since we applied a per-pixel classification accuracy assessment, spectral confusion between trees and crops could have been exacerbated by this [16]. In other words, some mono-cropped and inter-cropped maize fields could have had similar spectral features as a result of woody vegetation that can be found within maize fields. Also, in Machakos, the majority of the farmers cultivate crops around hamlets, and in many cases the cultivated fields are surrounded by pockets of natural vegetation [64]. However, we employed an empirical classification approach that could have been more robust in terms of practical and operational cropping system mapping. In addition, since our mapping results were produced using the most important variables (Figure 4), we assumed that collinearity is somewhat accounted for [65]. Furthermore, we tested the collinearity between the indices selected for final mapping (RE1_NDVI, RE1_NDRE, RE2_DVIedge, RE1_LCI and RE1_SIPE3) and found that they were not correlated.

Maize is generally vulnerable to numerous pests and diseases [66]. Choosing appropriate cropping systems can be a valuable alternative to the use of synthetic pesticides [67]. For instance, inter-cropping has been used as a buffer against the spread of plant pests and pathogens by attracting pests away from their host plant and also increasing the distance between plants of the same species, making it more exigent for the pest to target their main crop [68]. A good example is inter-cropping maize with cowpea/beans has been proven to reduce the maize stem borer [69] and cowpea/bean thrips [70] densities significantly. As a result, accurate baseline information on cropping systems could be used to better understand the relationship between cropping patterns and pest and disease propagation mechanisms such as the occurrence of the maize lethal necrosis (MLN) disease, first reported in Kenya in 2012, which is hypothesized to be linked to the spatial distribution of the cropping systems [71].

Essentially, “traditional” agricultural land use mapping often renders information on the spatial distributions or acreages of certain crops without further details of the actual underlying agronomical cropping systems [14]. Information on the cropping systems is vitally needed as a spatial descriptor (parameter) within commonly used crop modeling schemes such as the Decision Support System for Agrotechnology Transfer (DSSAT), since these crop systems are key determinants for agricultural production and food supply, given that mono-cropped systems generally may exhibit different yield cycles than mixed cropping systems. With the advent of new satellite constellations with better pixel and temporal resolution, not only crop mapping but also crop systems characterization can be performed. This will be of great use to crop scientists and decision makers. Moreover, cropping patterns can be related to climate change effects and thus to agricultural productivity, and as a result the extent of food security as, for instance, mixed cropping systems are a key adaptation mechanism for areas experiencing considerable climate variability.

## 6. Conclusions

This study evaluated the potential of 5-m RapidEye multispectral data and the advanced RF classification technique in mapping maize cropping systems in a complex, dynamic and heterogeneous landscape. To the best of our knowledge, we produced the first cropping patterns map for maize-based cropping systems in Kenya. We conclude that RapidEye imagery acquired during stem elongation and flowering phenological development stages give satisfactory results for separating mono- and mixed maize cropping systems.

We suggest that data on cropping systems mapping, using high resolution time-series data, are useful baseline information feeds to monitor and understand seasonal cropping pattern changes as a function of climatic variability and climate change in Africa. This information, especially if linked with yields and crop pest and disease infestation levels may be very useful for better agricultural risk projections. Upholding the widely recognized role of small-scale farming for food security in Africa, the extent, distribution and dynamics of cropping systems, as one of the important variables in crop productivity models, should further be investigated. This will result in increased farm sizes hence making it more feasible to monitor cropping systems using freely available remote sensing data sets.

However, temporal availability of high resolution data is still restricted by the high cost of imagery from semi-commercial sensors such as RapidEye and frequent cloud cover, especially in the tropics. Upscaling the results from this study to wide-area monitoring of cropping patterns is thus still challenging. However, freely available multi-temporal data from Landsat-8 combined with Sentinel 2a and Sentinel 2b data should be further investigated and exploited to improve cropping systems mapping in Africa and beyond. Overall, the relatively accurate classification results obtained in this study provide dependable information that could be used to complement region or field-specific yield data to aid decision making in terms of improved crop productivity and food supply management.

## Figures and Tables

**Table 1:** Spectral vegetation indices used in the study. Source information is given in the last column.

Name	Index	Formula	Reference
Canopy Chlorophyll Content Index	CCCI	$\left((R_{\mathrm{NIR}}-R_{\mathrm{red}\_\mathrm{edge}})/\left(R_{\mathrm{NIR}}+R_{\mathrm{red}\_\mathrm{edge}}\right))/\left((R_{\mathrm{NIR}}-R_{\mathrm{red}}\right)/\left(R_{\mathrm{NIR}}+R_{\mathrm{red}}\right)\right)$	[28]
Normalized Difference Red-Edge	NDRE	$(R_{\mathrm{NIR}}-R_{\mathrm{red}\_\mathrm{edge}\;})/(R_{\mathrm{NIR}}+R_{\mathrm{red}\_\mathrm{edge}}$)	[29]
Transformed Soil Adjusted Vegetation Index	TSAVI	$B(R_{\mathrm{NIR}}-{B*R}_{\mathrm{red}}-A)/(R_{\mathrm{red}}+\;B\left(R_{\mathrm{NIR}}-A\right)+X(1+B^{2}$))	[30]
Soil Adjusted Vegetation Index Red-Edge	SAVI-edge	$1.5*(R_{\mathrm{NIR}}-R_{\mathrm{red}\_\mathrm{edge}})/(R_{\mathrm{NIR}}+R_{\mathrm{red}\_\mathrm{edge}}+0.5$)	
Leaf Chlorophyll Index	LCI	$(R_{\mathrm{NIR}}-R_{\mathrm{red}\_\mathrm{edge}\;})/(R_{\mathrm{NIR}}+R_{\mathrm{red}}$)	[31]
Soil Adjusted Vegetation Index	SAVI	$1.5*(R_{\mathrm{NIR}}-R_{\mathrm{red}})/(R_{\mathrm{NIR}}+R_{\mathrm{red}}+0.5$)	[32]
Normalized Difference Vegetation Index	NDVI	$(R_{\mathrm{NIR}}-R_{\mathrm{red}\;})/(R_{\mathrm{NIR}}+R_{\mathrm{red}}$)	[33]
Difference Vegetation Index	DVI	$R_{\mathrm{NIR}}-R_{\mathrm{red}}$	[33]
Rationalized Normal Difference Vegetation Red-Edge Index	RNDVI-edge	$\left(R_{\mathrm{NIR}}-R_{\mathrm{red}}\right)/{\left(R_{\mathrm{NIR}}\;-R_{\mathrm{red}}\right)}^{1/2}$	[32]
Simple Ration	SR	$R_{\mathrm{NIR}}/{\;R}_{\mathrm{red}}$	[34]
Chlorophyll Green	Chlgreen	${\left(R_{\mathrm{NIR}}\;-R_{\mathrm{green}\;}\right)}^{-1}$	[35]
Chlorophyll Red-Edge	ChlRed-edge	${\left(R_{\mathrm{NIR}}-R_{\mathrm{red}\_\mathrm{edge}\;}\right)}^{-1}$	[35]
Green Normalized Difference Vegetation	GNDVI	$(R_{\mathrm{NIR}}-R_{\mathrm{green}\;})/(R_{\mathrm{NIR}}+R_{\mathrm{green}}$)	[36]
Simple Ratio 672/550 Datt5	SR672/550	$R_{\mathrm{red}}/{\;R}_{\mathrm{green}}$	[37]
Simple Ratio 695/670 Carter 5	Ctr5	$R_{\mathrm{red}\_\mathrm{edge}}/{\;R}_{\mathrm{red}}$	[38]
Simple Ratio 710/760 Carter 4	Ctr4	$R_{\mathrm{red}\_\mathrm{edge}}/{\;R}_{\mathrm{NIR}}$	[38]
Wide Dynamic Range Vegetation Index	WDRVI	$(0.1R_{\mathrm{NIR}}-R_{\mathrm{red}\_\mathrm{edge}\;})/(0.1R_{\mathrm{NIR}}+R_{\mathrm{red}\_\mathrm{edge}}$)	[39]
Enhanced Vegetation Index	EVI	$2.5*(R_{\mathrm{NIR}}-R_{\mathrm{red}})/(R_{\mathrm{NIR}}+2.4R_{\mathrm{red}}$+1)	[39]
Modified Chlorophyll Absorption Ratio Index	MCARI	$((R_{\mathrm{NIR}}-R_{\mathrm{red}\;})-0.2\left(R_{\mathrm{red}\_\mathrm{edge}}+R_{\mathrm{green}}\right)$)(${\;R}_{\mathrm{red}\_\mathrm{edge}}/{\;R}_{\mathrm{red}})$	[40]
Rationalized Normal Difference Vegetation Index	RNDVI	$\left(R_{\mathrm{NIR}}-R_{\mathrm{red}\_\mathrm{edge}}\right)/{\left(R_{\mathrm{NIR}}-R_{\mathrm{red}\_\mathrm{edge}}\right)}^{1/2}$	
Disease Water Stress Index	DSWI-4	$R_{\mathrm{green}}/{\;R}_{\mathrm{red}}$	[41]
Modified Chlorophyll Absorption Ratio Index	MCARI		
Structure Intensive Pigment Index 3	SIPI3	$(R_{\mathrm{NIR}}-R_{\mathrm{blue}\;})/(R_{\mathrm{NIR}}+R_{\mathrm{red}}$)	[42]
Anthocyanin Reflectance Index	ARI-edge	$\left(1/{\;R}_{\mathrm{green}\;}\right)-(1/{\;R}_{\mathrm{red}\_\mathrm{edge}}$)	[43]
Disease Water Stress Red-edge Index	DSWI-edge		
Structure Intensive Pigment Index 2	SIPI2	$(R_{\mathrm{NIR}}-R_{\mathrm{blue}\;})/(R_{\mathrm{NIR}}+R_{\mathrm{red}\_\mathrm{edge}}$)	[42]
Enhanced Vegetation Index Red-Edge 2	EVI-edge 2	$2.5*(R_{\mathrm{NIR}}-R_{\mathrm{red}\_\mathrm{edge}})/(R_{\mathrm{NIR}}+\;2.4R_{\mathrm{red}\_\mathrm{edge}}+$1)	
Transformed Soil Adjusted Vegetation Index Red-Edge	TSAVI-edge	$B(R_{\mathrm{NIR}}-{B*R}_{\mathrm{red}\_\mathrm{edge}}-A)/(R_{\mathrm{red}\_\mathrm{edge}}+\;B\left(R_{\mathrm{NIR}}-A\right)+X(1+B^{2}$))	
Difference Vegetation Index Red-Edge	DVI-edge	$R_{\mathrm{NIR}}-R_{\mathrm{red}\_\mathrm{edge}}$	
Green Leaf Index	GLI	$2(R_{\mathrm{green}}-R_{\mathrm{red}}-R_{\mathrm{blue}})/2(R_{\mathrm{green}}+R_{\mathrm{red}}+R_{\mathrm{blue}}$)	[40]

**Notes:** R_blue_, R_green_, R_red_, R_red_edge_ and R_NIR_ are surface reflectance value at blue (band 1) green (band 2), red (band 3), red-edge (band 4) and near infrared (band 5) of RapidEye. The parameters for Transformed Soil Adjusted Vegetation Index (TSAVI) slope of the soil line (A) = 1.2, intercept of the soil line (B) = 0.04 and adjustment factor(X) = 0.08.

**Table 2:** Overall accuracies and kappa coefficient of agreement for the Land Use Land Cover classification.

Analysis	Overall Accuracy (%)	Kappa Coefficient
RE (bands)	87.46	0.86
RE (bands) + All RE_veg indices	86.41	0.84
RF selected spectral variables	93.20	0.91

**Table 3:** Random forest classification confusion matrix for the land use/land cover classes (level 1) using the 15 most important RapidEye spectral variables and 30% of the reference data.

Class	Artificial Surface	Cropland	Natural Vegetation	Water Bodies	Total	UA (%)	F1 Score
Artificial Surface	904	23	0	18	945	96.48	0.96
Cropland	11	845	89	0	945	87.84	0.89
Natural Vegetation	0	94	851	0	945	90.53	0.90
Water Bodies	22	0	0	923	945	98.09	0.98
Total	937	962	940	941	3780		
PA (%)	95.66	89.42	90.05	97.67			
OA (%)	93.20						

**Table 4:** Overall accuracies and kappa coefficient of agreement for the two maize mono- and mixed cropping systems.

Analysis	Overall Accuracy (%)	Kappa Coefficient
RE (bands)	80.24	0.77
RE (bands) + All RE_veg indices	73.38	0.70
RF selected spectral variables	85.71	0.84

**Table 5:** Random forest classification confusion matrix for mapping cropping systems using the most important 15 RapidEye spectral variables and 30% of the reference data.

Class	Mono Cropping	Mixed Cropping	Total	UA (%)
Mono maize cropping	486	74	560	84.97
Mixed maize cropping	86	474	560	86.50
Total	572	548	1120	
PA (%)	86.79	84.64		
OA (%)	85.71			
QD (%)	1.00			
AD (%)	13.00			
